# Evaluation of Functional Connectivity in the Brain Using Positron Emission Tomography: A Mini-Review

**DOI:** 10.3389/fnins.2019.00775

**Published:** 2019-07-26

**Authors:** Tadashi Watabe, Jun Hatazawa

**Affiliations:** ^1^Department of Nuclear Medicine and Tracer Kinetics, Graduate School of Medicine, Osaka University, Osaka, Japan; ^2^Institute for Radiation Sciences, Osaka University, Osaka, Japan

**Keywords:** PET, functional connectivity, default mode network, blood flow, metabolism

## Abstract

Resting-state networks (RSNs) exhibit spontaneous functional connectivity in the resting state. Previous studies have evaluated RSNs mainly based on spontaneous fluctuations in blood oxygenation level-dependent (BOLD) signals during functional magnetic resonance imaging (fMRI). However, separation between regional increases in cerebral blood flow (CBF) and oxygen consumption is theoretically difficult using BOLD-fMRI. Such separation can be achieved using quantitative ^15^O-gas and water positron emission tomography (PET). In addition, ^18^F-FDG PET can be used to investigate functional connectivity based on changes in glucose metabolism, which reflects local brain activity. Previous studies have highlighted the feasibility and clinical usefulness of ^18^F-FDG-PET for the analysis of RSNs, and recent studies have utilized simultaneous PET/fMRI for such analyses. While PET provides seed information regarding the focus of the abnormalities (e.g., hypometabolism and reduced target binding), fMRI is used for the analysis of functional connectivity. Thus, as PET and fMRI provide different types of information, integrating these modalities may aid in elucidating the pathological mechanisms underlying certain diseases, and in characterizing individual patients.

## Introduction

Resting-state networks (RSNs) exhibit spontaneous functional connectivity in the resting state. Among these is the default mode network (DMN), in which the posterior cingulate cortex (PCC) plays a central role, connecting with the medial-prefrontal, hippocampal, and lateral temporal areas (Raichle et al., 2001; Buckner et al., 2008; Raichle, 2015). While the DMN is active during the resting state, it becomes deactivated during task states (McCormick and Telzer, 2018). Previous studies have demonstrated that the DMN is related to cognitive functions including memory, thinking, and the integration of social information. Furthermore, DMN abnormalities have been observed in patients with autism, schizophrenia, and Alzheimer’s disease (AD) (Buckner et al., 2008).

The default mode network was first discovered by Raichle et al. using positron emission tomography (PET) (2001). The authors focused on the regional oxygen extraction fraction (OEF), and which refers to the ratio of the cerebral metabolic rate of oxygen (CMRO_2_) to cerebral blood flow (CBF). Increases in CBF during task-related brain activation occur in conjunction with smaller changes in CMRO_2_, resulting in a relative decrease in the OEF. In contrast, the OEF increases during the deactivated state. Raichle et al. (2001) reported that the OEF increases in the visual cortex of healthy adults during the resting state (i.e., awake with eyes closed). Eye closure usually induces deactivation in the visual cortex and corresponding increases in OEF. However, many brain regions exhibit decreases in OEF when compared to the mean hemispheric value, suggesting that some areas are activated even in the baseline or “default” state of the brain.

Since the discovery of the DMN, several studies have used functional magnetic resonance imaging (fMRI) to analyze functional connectivity based on spontaneous fluctuations in blood oxygenation-level dependent (BOLD) signals (Greicius et al., 2003). When an area of the brain is activated, the BOLD signal of the corresponding region increases, reflecting a larger increase in regional CBF than in regional CMRO_2_ (Raichle et al., 2001). Therefore, functional connectivity among brain regions can be estimated by evaluating the correlations among the time-courses of changes in BOLD signals. While the methodologies for detecting decreased OEF on PET and increased BOLD signaling on fMRI are similar, PET is associated with superior quantitative accuracy for the evaluation of CBF and CMRO_2_, which are determined separately in the calculation of OEF.

In the present study, we summarized recent advances in the evaluation of functional connectivity using PET. Since the methodological aspects of connectivity analysis have been summarized by Aiello et al. (2016), Yakushev et al. (2017), and Verger and Guedj (2018), we mainly focused on how PET data beyond fluorodeoxyglucose (FDG) can be utilized for functional connectivity analyses. Using the term “functional connectivity PET,” we searched PubMed for articles published between February 2016 and February 2019. Similarly, we searched PubMed using the term “functional connectivity FDG PET” for articles published between February 2014 and February 2019. We included clinical studies involving patients or healthy volunteers in which PET was used for connectivity analyses. Preliminary studies and those with incomplete methodological information were excluded from our analysis.

## Evaluation of Functional Correlations Using ^15^O-gas PET

**FIGURE 1:**
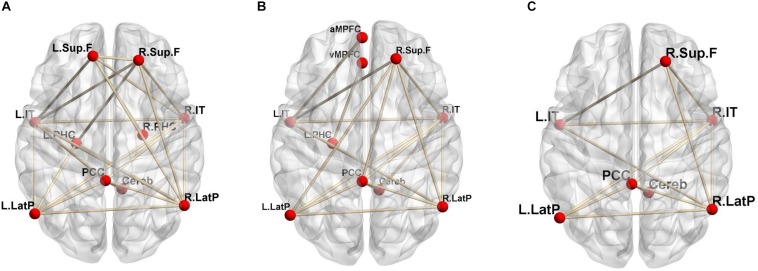
Significant positive functional correlations involving the DMN. **(A)** Correlations based on CBF; **(B)** CMRO_2_; **(C)** the overlap between CBF and CMRO_2_ (reprint from Aoe et al., 2018; permission obtained from the *Annals of Nuclear Medicine* in accordance with their open access policy).

Regional CBF and CMRO_2_ can be quantitatively evaluated via ^15^O-labeled gas and water PET (Hatazawa et al., 1996; Watabe et al., 2014). Our group recently reported that functional correlations in the DMN can be estimated using quantitative ^15^O-PET (Aoe et al., 2018). Our findings indicated that evaluation based on changes in CBF revealed a larger number of brain networks than evaluation based on changes in CMRO_2_, suggesting that the contribution of blood flow to functional correlations in the DMN is greater than that of oxygen consumption (Figure 1). In this method, two brain regions are considered functionally correlated when their quantitative CBF or CMRO_2_ values exhibit a significant positive inter-subject correlation. Similar methods were also adopted in another previous study (Di et al., 2012).

**FIGURE 2:**
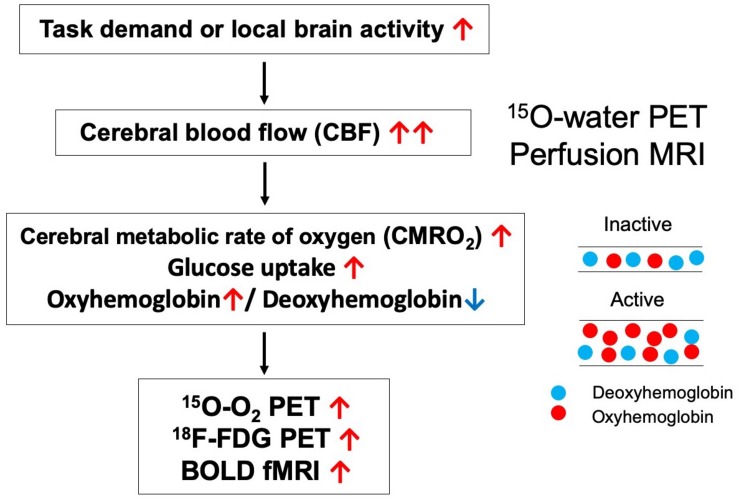
Brain responses during activation for each modality.

Unlike BOLD-fMRI, ^15^O-gas and water PET is advantageous in that CBF, and CMRO_2_ can be evaluated separately. Local brain activation first increases regional CMRO_2_, reflecting increases in energy metabolism, following which CBF increases (Figure 2). However, one disadvantage of PET is that it is difficult to clarify temporal changes due to the long scan duration of each acquisition and the time required for radioactive decay when compared with fMRI, which exhibits a temporal resolution on the order of seconds. Therefore, researchers should remain aware that the theoretical bases of determining functional correlations via PET and determining functional connectivity via BOLD-fMRI are different.

## Evaluation of Functional Connectivity Via ^18^F-FDG PET

Glucose is the fundamental metabolic substrate in the brain. Given that glucose metabolism reflects local brain activity, this measure can be used to analyze functional connectivity. Passow et al. (2015) reported a close association between local metabolic activity and functional connectivity by comparing fluctuations in FDG-uptake and BOLD signals, suggesting that ^18^F-FDG-PET is feasible and reliable for functional connectivity analyses in the brain. In their study, FDG-PET images were acquired in 12 frames (5 min each) following intravenous injection of FDG, and fluctuations among these frames were evaluated based on global normalization. Wehrl et al. (2013) also utilized temporal information from dynamic PET, while additional studies have highlighted the utility of static PET data for the evaluation of metabolic functional connectivity. In a graph theory-based network analysis, Vanicek et al. (2016) evaluated preoperative FDG-PET data for patients with right-sided temporal lobe epilepsy (TLE) (RTLE; *n* = 30), left-sided TLE (LTLE; *n* = 32), and healthy controls (*n* = 31). FDG-PET has also been used in clinical practice to detect seizure lateralization in patients with TLE. Indeed, patients with RTLE exhibit higher lobar connectivity weights than those with LTLE for connections involving the temporal and parietal lobes of the contralateral hemisphere. Although the mechanisms underlying such differences remain to be fully elucidated, the authors suspected that compensatory mechanisms were more prominent in patients with RTLE than in those with LTLE.

Pagani et al. (2017) performed independent-component analysis of ^18^F-FDG PET data, revealing that patients with AD exhibit gradual disruptions in functional brain connectivity during the progression of cognitive decline. Chen et al. (2018) investigated alterations in whole-brain intrinsic functional connectivity in patients with dementia with Lewy Bodies (DLB). In their study, the DLB group exhibited increases in the strength of some connections when compared to the healthy control group. These findings suggest that alterations in functional connectivity can be evaluated with a certain level of sensitivity in patients with dementia using ^18^F-FDG PET.

Another study highlighted the usefulness of functional connectivity analysis for predicting responses to vagus nerve stimulation (VNS) in patients with epileptic seizures (Yu et al., 2018). Yu et al. (2018) performed preoperative FDG-PET in pediatric patients with refractory epilepsy undergoing VNS and analyzed metabolic connectivity via an independent component analysis. The authors reported significant differences in metabolic connectivity between the VNS-effective and VNS-ineffective groups. Relative changes in glucose metabolism were strongly connected among areas of the brainstem, cingulate gyrus, cerebellum, bilateral insula, and putamen in the VNS-effective group. Thus, these studies demonstrate that ^18^F-FDG-PET analyses of functional connectivity are clinically useful for identifying potential responders to therapy.

In, Villien et al., 2014 reported a new method for overcoming the temporal limitations of ^18^F-FDG-PET, observing that temporal changes in glucose metabolism can be evaluated during continuous injection of FDG. Since FDG is metabolically trapped in neurons or glial cells in the brain after being metabolized into glucose-6-phosphate by hexokinase, the upslope of the time-activity curves reflects real-time glucose metabolism. This technique can used to detect focal brain regions exhibiting task- or stimulus-related activation, similar to BOLD-fMRI (Jamadar et al., 2019). Although further technical adjustments are required to improve the analysis of functional connectivity during continuous-infusion PET, this technique is promising for overcoming the temporal limitations of PET.

## Integrated Detection Via PET and MRI

Recent studies have utilized simultaneous or combined PET/MRI for functional connectivity analysis, allowing for perfect co-registration in terms of spatial, and temporal matching (Table 1). Using an independent component analysis, Savio et al. (2017) compared the detection of RSNs between resting-state fMRI and ^18^F-FDG PET using simultaneous PET/MR in healthy volunteers (2017). Similar networks were detected using the two modalities, suggesting that coupling between glucose metabolism and blood oxygenation responses is preserved in healthy individuals. Marchitelli et al. (2018) reported that bioenergetic coupling between glucose utilization and neurovascular transmission decreases with age in healthy adults, and that more marked decreases are observed in patients with amnestic mild cognitive impairment (MCI) and mild-to-moderate AD (2018). In their study, ^18^F-FDG-PET was used to detect regions exhibiting hypometabolism, rather than for connectivity analysis. Within-subject analysis, in which correlations between glucose utilization (PET) and connectivity (fMRI) were evaluated for each participant, revealed that patients with MCI/AD exhibited significant decreases in such correlations. For between-subjects comparisons of PET/fMRI data, in which each imaging modality and metric (volumes) were concatenated across all participants, overlap between the two modalities varied depending on fMRI connectivity parameters.

**TABLE 1:** Main findings of functional connectivity analyses using PET and fMRI.

**Authors (year)**	**Participants**	**Method of analysis and main findings**
Savio et al., 2017	A total of 22 healthy, right-handed participants (15 men, 7 women; mean age: 54.5 ± 10.0 years)	**Acquisition:** Simultaneous PET/MRI with static ^18^F-FDG-PET. **Analysis:** ICA (20 components). **Results:** Similar RSNs were detected via fMRI and ^18^F-FDG PET when resting-state fMRI and ^18^F-FDG-PET data were acquired simultaneously.
Marchitelli et al., 2018	A total of 23 patients with aMCI/AD and 23 healthy older adults	**Acquisition:** Simultaneous PET/MRI with static ^18^F-FDG-PET. **Analysis:** Uptake on ^18^F-FDG-PET and resting-state fMRI metrics were compared between patients with aMCI/AD and controls and expressed as *Z* scores. **Results:** For within-subject PET/fMRI comparisons, correlations were high overall in healthy controls but 17% lower in patients with aMCI/AD (significant at *p* < 0.05). For between-subject comparisons, FDG/gICA-DR exhibited the greatest overlap around the posterior DMN nodes.
Yokoi et al., 2018	Amyloid-positive patients with early AD (*n* = 23) and amyloid-negative control participants (*n* = 24)	**Acquisition:** Separate PET (^18^F-THK5351, static) and MRI (3T). **Analysis:** Seed-based connectivity analysis was performed by generating seed ROIs based on regions exhibiting the most significant differences in ^18^F-THK5351 retention between patients with AD and controls. **Results:** Patients with AD exhibited significant decreases in connectivity between the PCC and widespread brain regions.
Kim et al., 2019	A total of 16 patients with MDD without comorbidities and 15 healthy controls	**Acquisition:** Separate PET [(^11^C)ABP688, dynamic] and MRI (3T). **Analysis:** The BP_ND_ of [^11^C]ABP688 was quantified using the SRTM for mGluR5 availability. Seed-based functional connectivity analysis was performed using resting-state fMRI data with regions derived from quantitative [^11^C]ABP688 PET as seeds. **Results:** Patterns of correlation between [^11^C]ABP688 BP_ND_ and the strength of functional connectivity involving the superior prefrontal cortex were opposite in the depression and control groups.
Hamilton et al., 2018	A total of 16 patients with MDD and 14 controls	**Acquisition:** Simultaneous fMRI and ^11^C-raclopride PET targeting the dopamine D_2_ receptor. **Analysis:** BP_ND_ was estimated via the MRTM, using the cerebellum as the reference tissue. Functional connectivity analysis was performed using striatal regions exhibiting significant between-group BP_ND_ differences as seeds. **Results:** Increased BP_ND_ and decreased connectivity were observed in the striatum. The BP_ND_ was increased in both the left ventral striatum and right dorsal striatum in patients with MDD. Connectivity between these regions and cortical targets was also decreased in the MDD group.

*ICA, independent component analysis; aMCI/AD, amnestic mild cognitive impairment/mild-to-moderate Alzheimer’s disease; gICA- DR, group independent component analysis with dual regression; PCC, posterior cingulate cortex; BP_*ND*_, non-displaceable binding potential; SRTM, simplified reference tissue model; mGluR5, metabotropic glutamate receptor-5; MRTM, multi-linear reference tissue model; MDD, major depressive disorder.*

## Utility of PET as a Seed for fMRI Analysis

Previous studies have compared the correlations between PET and fMRI data using simultaneous PET/MRI. Wehrl et al. (2014) proposed the concept of cometomics, which combines connectivity data and metabolic information. When comparing patients with healthy controls, regions exhibiting hypometabolism or reduced target binding on PET can be identified via statistical parametric mapping or region-of-interest (ROI)-based analyses. Resting-state BOLD-fMRI data can be used to derive RSN maps or related parameters during functional connectivity analyses. When combining these methods, regions exhibiting abnormalities on PET images are used as seed regions for the connectivity analysis.

Yokoi et al. (2018) performed resting-state fMRI and ^18^F-THK5351 PET in amyloid-positive patients with early AD (*n* = 23) and healthy, amyloid-negative controls (*n* = 24). To perform seed-based connectivity analysis, they generated several seed ROIs based on significant differences in ^18^F-THK5351 retention between the AD and control groups. Histological evaluations at autopsy indicated that ^18^F-THK5351 retention corresponded to tau deposition, monoamine oxidase-B (MAO-B) levels, and astrogliosis in the brains of patients with AD. Furthermore, seed-based connectivity analysis revealed significant decreases in connectivity between the posterior cingulate cortex (PCC) and widespread brain regions in patients with AD.

Kim et al. (2019) performed seed-based functional connectivity analysis using resting-state fMRI data with seed regions derived from quantitative [^11^C]ABP688 PET analysis in patients with major depression. To determine metabotropic glutamate receptor-5 (mGluR5) availability, the binding potential (BP_ND_) of [^11^C]ABP688 was quantified using the simplified reference tissue model. ROI-based analysis revealed that the [^11^C]ABP688 BP_ND_ in the prefrontal cortex was significantly lower in patients than in controls. BP_ND_ seed-based functional connectivity analysis revealed significantly reduced connectivity between the inferior parietal cortex and fusiform gyrus/inferior occipital cortex in patients, relative to that observed in controls. Patterns of correlation between [^11^C]ABP688 BP_ND_ and the strength of functional connectivity involving the superior prefrontal cortex seed were opposite in the depression and control groups. Thus, using PET to determine mGluR5 availability revealed related alterations in functional connectivity in the patient group. In addition, Hamilton et al. (2018) conducted whole-brain functional connectivity analysis via fMRI using striatal regions exhibiting significant BP_ND_ differences on ^11^C-raclopride PET as seeds. They examined dopamine D_2_ receptors using ^11^C-raclopride PET in patients with major depressive disorder (*n* = 16) and controls (*n* = 14). Increased BP_ND_ in both the left ventral striatum and right dorsal striatum and decreased functional connectivity between the affected striatal areas and their respective cortical targets were observed in patients with major depressive disorder.

Although previous reports on functional connectivity determined using simultaneous PET/MR are still limited (Table 1), a combination of connectivity data and metabolic/receptor information will be the major approach in future research.

## Conclusion

Functional correlations between blood flow and oxygen consumption can be separately estimated using quantitative ^15^O-gas and water PET. Glucose metabolism during ^18^F-FDG PET can also be used for functional connectivity analysis. Moreover, PET can provide information regarding the focus of abnormalities, which can then be used for functional connectivity analyses via fMRI. Comparisons between PET and fMRI data may thus allow for improved understanding of the pathophysiology of several brain disorders. Such comparisons may also aid in characterizing individual patients with these disorders.

## Author Contributions

Both authors wrote and approved the final version of the manuscript.

## Conflict of Interest Statement

The authors declare that the research was conducted in the absence of any commercial or financial relationships that could be construed as a potential conflict of interest.
